# Modeling COVID-19 Transmission Dynamics: A Bibliometric Review

**DOI:** 10.3390/ijerph192114143

**Published:** 2022-10-29

**Authors:** Gour Gobinda Goswami, Tahmid Labib

**Affiliations:** Department of Economics, North South University, Dhaka 1229, Bangladesh

**Keywords:** COVID-19, COVID-19 transmission, bibliometric review, public health, infectious diseases

## Abstract

A good amount of research has evolved just in three years in COVID-19 transmission, mortality, vaccination, and some socioeconomic studies. A few bibliometric reviews have already been performed in the literature, especially on the broad theme of COVID-19, without any particular area such as transmission, mortality, or vaccination. This paper fills this gap by conducting a bibliometric review on COVID-19 transmission as the first of its kind. The main aim of this study is to conduct a bibliometric review of the literature in the area of COVID-19 transmission dynamics. We have conducted bibliometric analysis using descriptive and network analysis methods to review the literature in this area using RStudio, Openrefine, VOSviewer, and Tableau. We reviewed 1103 articles published in 2020–2022. The result identified the top authors, top disciplines, research patterns, and hotspots and gave us clear directions for classifying research topics in this area. New research areas are rapidly emerging in this area, which needs constant observation by researchers to combat this global epidemic.

## 1. Introduction

The first cases of COVID-19 were first reported in December 2019. On 12 December 2019, a cluster of patients in Wuhan began to experience pneumonia symptoms but did not respond to standard treatment. It was the first reported incident of COVID-19. Later, on 10 January 2020, the World Health Organization announced that the outbreak in Wuhan, China, was caused by the 2019 Novel Coronavirus (2019-nCoV) [1]. Then, the virus started traveling from one part of the world to another. New variants have been added to the list [2]. Wave after wave appeared on the scene, taking away the lives of millions of people worldwide and putting the whole world under a severe recession in the aftermath [3,4]. Scientists have started working on it in the following areas: the causes of this virus, the determinants and magnitudes of transmission, the determinants of death or mortality, and the determinants of vaccination, its hesitancy, and inequality in adoption. Some papers are exclusively dedicated to studying this pandemic’s social, political, environmental, ecological, and economic impact. It is difficult to find a discipline not being affected by COVID-19 research. The literature has evolved so fast that dozens of articles are published daily in all ranks of journals, including some top journals. Many reputed journals are also posting special issues on COVID-19. The research is so impactful that a broad body of literature has also evolved in an interdisciplinary fashion. Taking stock of all the essential works and a comparative study concerning their role in knowledge creation and dissemination is crucial.

Since the outbreak of this pandemic, a good number of researchers all over the world have been involved in researching COVID-19 transmission dynamics. The virus has been rapidly mutating and changing its nature [2]. As a result, many scholarly publications have been on the evolution, mutation, transmission, contagiousness, and fatality of the COVID-19 virus. It is necessary to have a clear idea of how the literature has been evolving, the research trends and hotspots, what branch and sub-branch have been developed, and which areas of expertise contribute most to this research area intending to establish new research on this topic. It is essential for developing further research questions. However, due to the rapid increase in the number of studies in the literature and the development of new sub-branches, it is difficult for a researcher to obtain a clear idea about the recent developments. The conventional literature review process imposes a challenge. It might be inefficient to find the trends and developments in this research area because of the massive number of rapidly growing pieces of literature.

A systematic review [5], a meta-analysis [6], or a bibliometric analysis [7] can be used to review a large number of studies in the literature to find the general focus, pattern, and trends. Hence, this paper attempts to deal with this kind of review using bibliometric analysis, which is primarily data-driven and evidence-based.

This paper aims to conduct a bibliometric literature review on COVID-19 transmission-related research papers published between 2020 and 2022. The endpoint is 13 June 2022. It is hoped that this kind of review will help researchers in this area to obtain quick and hands-on information about the latest research enterprise and its trends and explore the possible research gaps to formulate new research projects. For this purpose, this paper tries to answer the following questions:

Q1. Researchers from which areas of expertise or knowledge domain have studied the COVID-19 transmission dynamics.
Q2. What sources (Journals, Books, Conferences, etc.) might be relevant for further study of COVID-19 transmission dynamics?
Q3. Who have been the most productive authors in this research area?
Q4. What are the most impactful papers in this research area?
Q5. Which countries are leading in conducting research in this area?
Q6. What is the intellectual structure manifested in discipline orientation?
Q7. What have been the research hotspots and research developments in this research area?

A few bibliometric reviews have already been conducted in the literature [8,9,10,11,12,13,14,15], especially in the general theme of COVID-19 without any particular area such as transmission, mortality, and vaccination. This paper fills this gap by conducting a bibliometric review on COVID-19 transmission as the first of its kind. It is to be mentioned that the transmission channel is the most widely researched area among all the significant aspects of this pandemic because it involves science, politics, governance, culture, and all aspects of human development.

Pritchard first used the term bibliometrics in 1969. Bibliometric analysis is a way of analyzing bibliographic information from a collection of documents using mathematics and statistics. This method aims to measure research productivity by examining the publication trends and valuing its impact based on some metrics [16]. This method can also be applied to discover the collaboration pattern of a specific field of study, research hotspots, and collaboration networks through visualizing and mapping the literature on particular network analyses [17].

A similar type of method is systematic review and meta-analysis. A systematic review collects all possible studies based on a preset criterion, such as research questions, research area, and research design. This collection is later used for reviews and analysis of their results. However, these methods appear to be limited in presenting biased or poor-quality findings [18,19]. To avoid this, a meta-analysis is generally conducted on randomized controlled trials with solid evidence [20].

This study aims to find the research trend, hotspots, relevant sources, and authors in COVID-19 transmission dynamics. A systematic review and meta-analysis analyze the results of different studies on similar topics. On the other hand, bibliometric analysis deals with bibliographic data to find research productivity and patterns in the field or particular study area. With this end in view, Section 2 puts forward the methodology followed by the results and interpretation in Section 3. Section 4 provides a discussion, while Section 5 concludes this paper.

## 2. Research Methodology

### 2.1. Data

The Scopus citation index database was retrieved as the source for this study. The retrieval search query was “TITLE-ABS-KEY ((“COVID-19 *” OR “SARS-CoV-2”) AND (“transmission model*” OR “transmission dynamic model” OR “transmission dynamics”))”. The database was accessed on 13 June 2022. A total of 1103 pertinent publications were collected. The result was saved as “.csv” with “Citation Information”, “Bibliographical Information”, and “Abstract & Keywords”. Our dataset contained the following types of documents (Table 1).

The type of documents is dominated by articles mostly. Out of 1103 documents, only small fractions represent other types of work such as book chapters, conference papers, or reviews. The average citation per document is 23.75. This sample was later used to conduct the bibliometric analysis.

### 2.2. Analytical Tools

To analyze and visualize the data, the following software was used: (i) RStudio (Version 4.4.1), (ii) Openrefine (Version 3.5.2), (iii) VOSviewer (Version 1.6.17), and (iv) Tableau (Version 2022.2). The “Bibliometrix” [21] package in R studio was utilized. The Bibliometrix package is designed for analyzing bibliographic data. This package allows for descriptive analysis and network analysis. Openrefine [22] is an open resource software used for cleaning the data. VOSviwer [23] is another open resource software based on the VOS technique used to visualize bibliographic networks and knowledge maps. Tableau [24] is another data visualization software, especially for producing maps.

### 2.3. Author’s Co-Citation Analysis

Co-citation analysis is one of the most common methods used in the bibliometric analysis. When two documents are cited together in an article, it is known as a co-citation. If two documents are cited together frequently by other articles/documents, they are likely thematically similar [25]. One of the widely used citation analyses is author co-citation analysis. It is a way to identify the intellectual structure of the knowledge domain. According to Shafique (2013), “The intellectual structure refers to the knowledge fundamental of the examined scientific domain, representing some attributes, including its disciplinary composition, influence research topics, and the patterns of its interrelationships” [26]. It can also be utilized to trace and visualize the scholarly network of a study area [27]. Disciplines such as marketing, management information system, family business research, and tourism successfully used co-citation analysis to map the intellectual structure [28].

### 2.4. Keyword Co-Occurrence Analysis

Keyword co-occurrence analysis is a popular co-word method used nowadays [29]. As with citation analysis, it assumes that words often occurring together are thematically similar. It is helpful to study a large number of documents. This method helps to generate clusters and summarize the diverse research areas of a specific field of study [30]. This summary describes the internal relationship and structure in a specific field and guides the researcher in finding the core content of the literature. This study utilizes the author’s keyword to create a keyword co-occurrence network. One problem with using an author’s keyword is that different authors use different keywords to describe the same meaning. Thus, it is necessary to standardize the keywords [31]. In this paper, for example, (i) COVID-19, (ii) Novel Coronavirus, (iii) SARS-CoV-2, and (iv) Severe acute respiratory syndrome coronavirus 2 were standardized to COVID-19. Openrefine was used to standardize the keywords. After standardization, 1939 keywords were produced. Among these 1939 keywords, only 2% occurred a minimum of ten times, and 79% of keywords appeared only once.

### 2.5. Construction of Maps

A keyword co-occurrence network and co-citation analysis network are based on the Visualization of similarities, or the VOS, technique introduced by Nees Jan van Eck and Ludo Waltman [32]. The VOS technique utilizes a co-occurrence matrix to construct co-citation and co-word maps. The co-occurrence matrix is used to create a similarity matrix. It uses the association strength measure to create the similarity index. To create the similarity index, it uses the association strength measure [23].

Suppose that our similarity matrix is defined by $S_{ij}$, which denotes the similarity between item *i* and *j*. It must satisfy $S_{ij}>0,S_{ii}=0$ and $S_{ij}=S_{ji}$ for all $i,j\;\in \left\{1,\ldots ,n\right\}$. Using the association strength, the similarity between two items *i* and *j* are:

(1) $$S_{ij}=\frac{c_{ij}}{W_{i}W_{j}}$$

$c_{ij}$ = number of times item *i* and *j* co-occurred.

$W_{i}$ = number of times item *i* occurred.

$W_{j}$ = number of times item *j* occurred.

For example, counting the number of times any two keywords appear in the same documents, *n* keywords can build an *n* × *n* co-occurrence matrix. Later, using Equation (1), we can formulate the similarity matrix.

The VOS technique will create the map using the similarity matrix so that the distance between any pair of items *i* and j will reflect their similarity $S_{ij}$ as precisely as possible. Items with lower similarly will be located far from each other. Items with higher similarity will be located near each other and form a cluster of similar items. Moreover, the nodes’ size will indicate the number of times the items have occurred. A larger item node indicates that the item occurred more times than other items in the cluster.

To achieve clusters of similar items, the VOS mapping technique minimizes the weighted sum of the squared Euclidean distances between all pairs of items. The sum of the weighted squared distance will be higher if a pair of items have a higher similarity and vise-versa. The distance between each node can be calculated by the following equation:

(2) $$E\left(X;S\right)=\underset{i<j}{\sum }S_{ij}\|x_{i}-x_{j}\|{}^{2}$$

where ‖*‖ denotes the Euclidean norm. The n×k matrix X contains the location co-ordinates of each item, and k is the number of demotions in Euclidian space. Vector $x\_i=\left(x_{i1},\ldots ,x_{ik}\right)\;\in R^{k}$ denotes the ith row of X and contains the coordinates of object *i*.

In order to ensure no overlapping among items or to avoid solutions in which all items are at same co-ordinate, the following constraint is imposed:

$$\underset{i<j}{\sum }\|x_{i}-x_{j}\|=1$$

## 3. Results and Interpretation

### 3.1. Subject Area or Discipline-Wise Publications

The COVID-19 pandemic is not only a health issue. It has affected all aspects of human life, from society to the economy. As a result, the study of the transmission dynamics of COVID-19 is not limited only to the medical sector. Other disciplines are also equally suited for studying this area. The following table shows the number of studies conducted on different subject areas based on the classification provided by the Scopus database (Table 2):

Medicine has the most significant number of studies on COVID-19 transmission dynamics. Then, mathematics has the second most considerable number of studies. Mathematics is often used in medical studies. Mathematical applications can be used to identify the transmission source and medium of transmission and can even be used to estimate the number of infected people [33]. Biochemistry, Genetics, and Molecular Biology are the third most studied COVID-19 transmission dynamics. Molecular biology has revolutionized the identification of microorganisms through PCR testing and has provided information such as strain characteristics through genotyping [34]. Other subject areas that have studied the transmission dynamics of COVID-19 are Social Science, Neuroscience, Material Science, Pharmacology, Toxicology and Pharmaceutics, Economics, Econometrics, and Finance. Due to the nature of the topic, many publications under the COVID-19 transmission study are considered studies from multiple subject areas. For example, mathematical models of COVID-19 transmission dynamics are regarded as a mathematical and epidemiological study. We consider only the top ten subjects in Table 2. Other smaller categories are included in other categories.

### 3.2. Most Relevant Sources

In this dataset, there are 463 sources from a variety of disciplines (Table 3). Based on the number of publications in these various disciplines, the most relevant sources on COVID-19 transmission dynamics are selected. Among all of them, Scientific Reports dominates the publication side, followed by PLOS One. This also indicates that a good amount of research is being conducted in the pure health science area. This implies that other social sciences and business research should be encouraged to tackle transmission’s economic and social implications.

### 3.3. Top-Cited Publications

Top-cited publications also come from pure science and medical backgrounds, indicating medical advancement in this area within a short period. This kind of pattern may not have taken place in the past for other epidemics where the launching of vaccines took a long time, and it was sometimes successfully invented after the termination of the epidemic (Table 4).

### 3.4. Most Productive Authors (Sorted by Number of Articles)

The following table presents the most productive authors on COVID-19 transmission dynamics. Productivity is measured based on the number of articles produced by the author (Table 5). To address the quality issue, we also used the article fractionalization and H-index as other productivity indicators of productivity.

### 3.5. Country-Specific Publications

**Figure 1:**
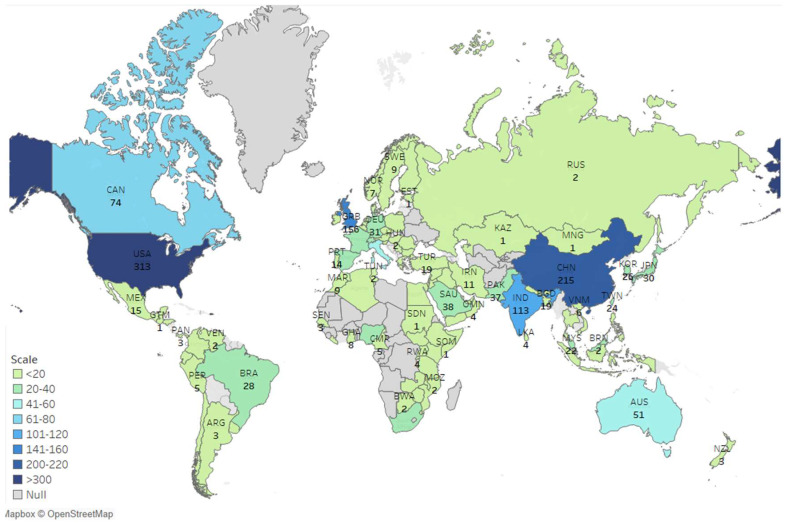
County–Specific productions (based on the Scopus database).

Finding the most productive countries will help us to determine which countries are leading the COVID-19 transmission dynamics research. Finding country-specific production is challenging. The bibliographic data do not come with any information regarding the country of origin of the documents. Nevertheless, we can use some proxies and try to measure the country-specific publications. The Scopus database provides a list of county-specific publications based on the search query. The index considers all the authors on a paper. The affiliation of each author is regarded as the country of origin. Thus, a single document is counted multiple times based on the number of authors. Based on the Scopus database, the following map is produced (Figure 1):

According to the list provided by Scopus, the most productive country is the USA, with 313 publications, followed by China, with 215 publications. Finally, the United Kingdom is the third most productive country in COVID-19 transmission dynamics research, with 156 publications.

**Figure 2:**
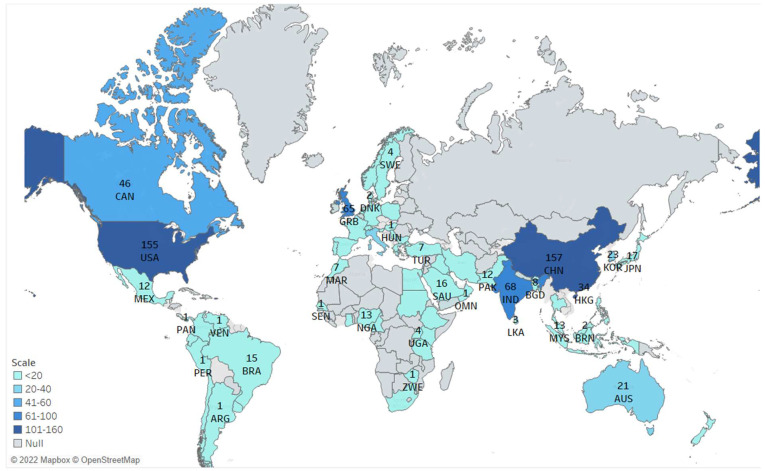
Country–Specific Production (Based on Corresponding Author).

The bibliographic dataset provides the list of corresponding authors (Figure 2). We created a country-specific production map based on the corresponding author’s affiliation. The corresponding author’s affiliation is considered the document’s country of origin, and a record is counted only once. In this case, we assume that the corresponding author is the lead author, and the corresponding author’s affiliation determines the publication’s origin. Studies have suggested that the corresponding authors contribute more to their article [35]; in most cases, the corresponding author is the lead author [36].

Based on the corresponding author’s affiliation, China has the most publications, followed by the USA, India, and the UK.

### 3.6. Co-Citation Network

**Figure 3:**
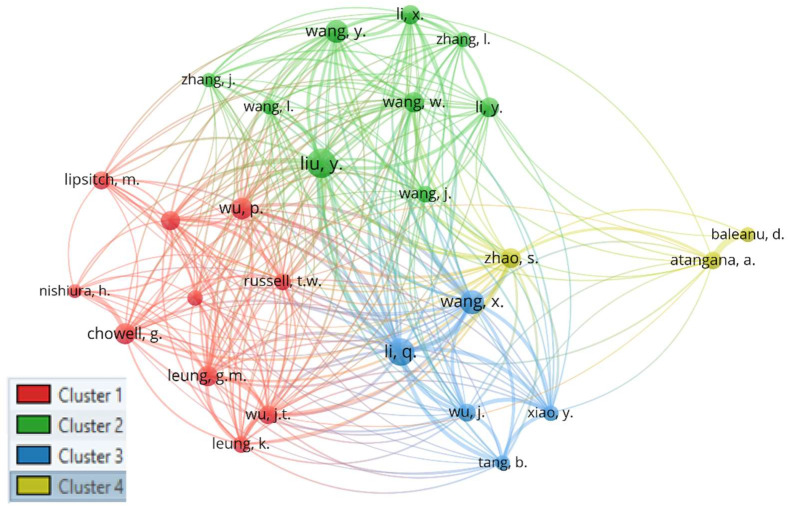
Co-citation network.

The following network presents the pattern of academic structure and relative distance between authors (Figure 3).

The distance between two authors in the network indicates the authors’ relatedness in terms of co-citation links. The network only shows authors who have been cited a minimum of 150 times. This threshold is set to keep the network relatively clean. That creates a network of 27 authors.

This network has four clusters. The authors’ research work [37,38,39,40] in the yellow cluster reveals that they are best known for their contributions to Mathematics. The red cluster includes authors best known for their work on the epidemiology of infectious diseases [41,42]. Most of the authors in this cluster are mathematicians, but they use their expertise to study the epidemiology of infectious diseases. The blue and green clusters primarily include authors affiliated with China, and their study areas are pretty similar. Authors in the blue cluster produced the early works on the COVID-19 study (i.e., Early Transmission Dynamics in Wuhan, China, Of Novel Coronavirus—Infected Pneumonia (2020). All of their work is based in China. Authors in the Green cluster primarily studied COVID-19 patients’ clinical characteristics, Pneumonia of COVID-19 patients, and the mortality rate of patients in China. Yongjin Li is the only author in the green cluster who worked on a mathematical analysis of China’s spread and control of the COVID-19 virus.

### 3.7. Co-Occurrence Network

**Figure 4:**
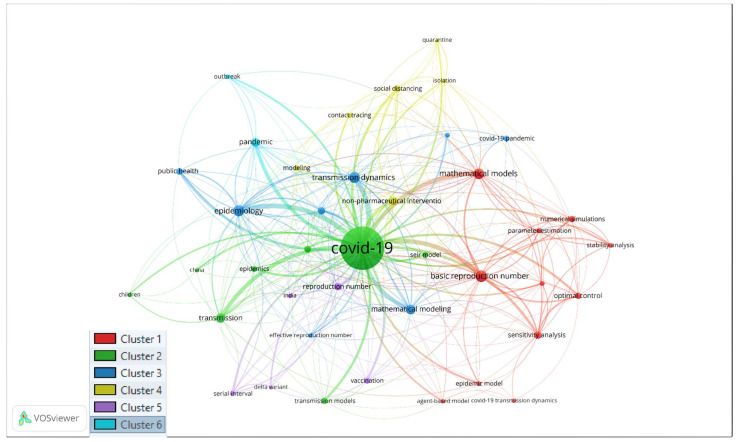
Keyword Co-occurrence.

Figure 4 shows the keyword co-occurrence network prepared using the VOS technique. There are six different clusters. Each cluster shows a different sub-field developed under the COVID-19 research area. The following table (Table 6) presents the keywords that appeared in different clusters.

### 3.8. Explaining Clusters (Table 5)

Cluster 1 (Red Cluster): Tools for studying the transmission dynamics of COVID-19

The co-occurring keywords include the agent-based model, basic reproduction number, COVID-19 transmission dynamics, epidemic model, mathematical models, numerical simulations, optimal control, parameter estimation, sensitivity analysis, stability, and stability analysis.

This cluster represents the tools that are used to model the transmission dynamics of COVID-19. The modeling of COVID-19 transmission dynamics is a type of epidemic modeling. Different mathematical models are used in epidemiological modeling, such as agent-based models, numerical simulations, and SEIR models. Tools such as sensitivity analysis and stability analysis are used to check the model’s robustness. It is worth noting that mathematical models are used not only in natural sciences such as physics, chemistry, or biology but also in social sciences, economics, and psychology. Mathematical models allow us to explain a system, study its components, and predict the system’s outcome. The concept of the total derivative will enable us to introduce the complex physical problems we encounter daily [38].

An agent-based model (ABM) is a computational model that simulates the action and interaction of agents with pre-specified characteristics in a pre-conditioned environment. The simulation helps to understand the system’s characteristics and what determines the outcome. ABM is widely used in the public health area. It has been adopted to study communicable and non-communicable diseases, health behaviors, and social epidemiology [43]. Many papers have adopted the ABM to check the transmission dynamics of COVID-19 [44,45,46].

The basic reproduction number or basic reproduction rate is generally denoted by $R_{0}$. This is defined as the average number of secondary cases that a primary case would generate in a susceptible population [47]. $R_{0}$ measures the ability of a new pathogen to spread. A higher value of $R_{0}$, greater than 1, indicates that the pathogen continues to spread if there is no environmental change or external intervention [48]. This measure is essential for disease control and mitigation effort [49]. Estimation of $R_{0}$ is conducted in different countries [50,51,52].

Numerical simulation is a complex calculation that is processed by the computer. This method is adopted when the behavior of a system is too difficult to model and interpret mathematically. Numerical simulation is another widely used tool in epidemiology. This technique is also used for modeling the model’s COVID-19 transmission dynamics and stability analysis [53,54]. A number of studies have used the SEIR model to estimate the transmission dynamics of COVID-19 [55].

Different interventions such as isolation, vaccination, and quarantine are required to control infectious diseases. However, the intervention needs to be administrated at the right time to eliminate the infectious disease. While modeling contagious diseases, optimal control theory has been proven to be an effective tool for introducing those interventions in the mathematical model to devise optimal disease intervention strategies [56]. Different studies have utilized numerical simulations and optimal control theory to model the transmission dynamics of COVID-19 [57,58,59].

Sensitivity analysis is a tool for measuring the robustness of an epidemic model. The objective of sensitivity analysis in an epidemic model is to test the influence of different parameters to find the most critical parameters [60] or to check how the observed result changes when other model parameters are changed [61].

Cluster 2 (Green): Children in COVID-19

The co-occurring keywords include children, China, COVID-19, COVID-19 disease, epidemic, SEIR model, transmission, and transmission model. This cluster represents the studies that focus on children. Children and older people have received special attention during the investigation of COVID-19 transmission dynamics because they seem to be the most vulnerable group in the population. Different transmission models were used to study the transmission dynamics of COVID-19 among children. Transmission models were also used to run simulations to predict and find patterns. The results help the researchers analyze the effectiveness of different strategies and suggest optimal strategies and interventions to policymakers.

Children could be dangerous spreaders. Their strong immune system might make them asymptomatic carriers. Several studies have focused on the epidemic characteristics of COVID-19 in children and their role as a spreader [62,63,64,65,66]. Studies have also found that vaccinating children could reduce the transmission of COVID-19 [67].

To reduce the transmission of COVID-19, most countries declared school closures. Various studies have suggested that school closure is ineffective in controlling COVID-19 [68]. The decision to reopen schools created a new question. Will children transmit COVID-19 to the household or family members? To answer this question, the transmission dynamics of COVID-19 in households or family clusters in the presence of children have been studied. The studies suggest that children are not likely to change transmission dynamics [69,70,71].

Cluster 3 (Deep Blue): Epidemiology

The co-occurring keywords include compartmental models, COVID-19 pandemic, effective reproduction number, epidemiology, infectious diseases, mathematical modeling, public health, and transmission dynamics. This cluster represents a specific field of study related to COVID-19 transmission dynamic analysis. Epidemiology analyzes the distribution, patterns, and determinants of health and disease conditions in a defined population [72]. This field of study is considered a cornerstone of public health and plays a vital role in policy decisions. One of the critical roles played by epidemiology is identifying the risk factors for diseases and suggesting preventive healthcare, especially in the case of infectious diseases [73].

Epidemiology plays a vital role in public health during the COVID-19 pandemic. Epidemiologists use different mathematical models (described in cluster 1) and genomic data to study the transmission dynamics, estimate the adequate reproduction number of COVID-19, and suggest the optimal time and strategies for preventing the transmission of COVID-19. Several studies have been conducted on the characteristics of the COVID-19 virus and its transmission dynamics, how the virus evolved, and comparing early and later variants.

Many studies have been conducted in genomic epidemiology to find the transmission dynamics of COVID-19 in different cities and lineages of the virus [74,75,76,77]. These studies can see how many different lineages are responsible for affecting each city or country and which lineages are more deadly, more transmissible, and faster. For example, one study estimated that lineage Gamma is 1.56 to 3.06 times more contagious than P.2 in Rio de Janeiro [74]. Moreover, the effective reproduction number of Gamma varies with geographic context. A similar type of study was conducted in India, Gujrat. They found more than 100 lineages of the virus, and this was associated with international travel [75].

There are also a good number of studies on the physical structure of the virus, how the virus evolved, and finally, how the virus changes over time. Studies have suggested that the COVID-19 virus presents low genetic diversity but has an increasing trend and several hotspot mutations throughout its genome [78,79]. Studying the virus’s physical and protein structure is essential for developing vaccines and therapeutics. Thus, some researchers have studied the virus’ physical and protein structure in the early stages of COVID-19 transmission dynamics research [80,81,82,83].

Cluster 4 (Yellow Cluster): Prevention Measure of COVID-19

The co-occurring keywords include contact tracing, isolation, modeling, non-pharmaceutical intervention, quarantine, and social distancing. This cluster represents the study of non-pharmaceutical interventions to prevent the spread of COVID-19. Non-pharmaceutical interventions (NPIs) refer to actions taken by people and the community to stop the spread of an infectious disease apart from becoming vaccinated or taking medicine [84]. These actions include isolating infected people, finding potentially infected persons through contact tracing and quarantining them to prevent community transmission, and maintaining social distance to reduce community transmission. These actions are considered the pillar of controlling COVID-19 [85].

Isolation means separating sick people with a contagious disease from people who are not ill [86]. Isolation is often performed in a hospital setting. In exceptional cases, it can be done at home [87]. For example, the USA and China transformed hospitals’ parking areas or built special isolation facilities.

Quarantine is the most effective and oldest tool used by people to control the epidemic. The recorded history of quarantine was found in fourteenth-century Italy [88]. Quarantine restricts the movement or separates healthy persons who have been exposed to the contagious disease but are not ill, because either they are not infected or still in the incubation period or are asymptomatic [89]. Quarantining exposed people prevents community transmission of the disease.

A combination of isolation and quarantine has been proven to be an effective strategy for preventing secondary transmission of COVID-19 [90,91,92,93,94].

Contact tracing is a public health practice that is used to identify and notify people who have been exposed to contagious diseases. To prevent the spreading, it is also necessary to identify the potential COVID-19 patients, quarantine them, and keep them under observation. Studies have found mixed results regarding the effectiveness of contact tracing in slowing down the spread of COVID-19 and death due to COVID-19. Some studies have suggested that it is effective [95,96,97], and others have suggested that it is not effective [98].

Theoretically, social distancing has been proven to be an effective strategy to slow down the spread of COVID-19 from an economic and epidemiological perspective [99,100]. Many countries successfully took social distancing as a preventive strategy to slow down secondary transmission.

Cluster 5 (purple cluster): Study of Delta variant

The co-occurring keywords include delta variant, India, serial interval, reproduction number, and vaccination. This cluster represents research on the delta variant of COVID-19 and COVID-19 research in India. India is the second most populated country in the world, with one of the lowest GDP per capita and a poor health system. India’s per-case mortality rate was 1.2%, one of the highest in the world. Thus, many studies were conducted in India to find out how one of the most populated counties with inferior healthcare systems should plan its strategy to fight COVID-19. These studies include finding the transmission dynamics of different COVID-19 variants using various mathematical models and simulations, the reproduction number of the virus, and predicting the number of possible infections [101,102,103]. Indian researchers widely used the “wastewater surveillance” technique to detect coronavirus in the environment [104].

The delta variant was first reported in India [105]. Thus far, it was one of the deadliest and most infectious variants of COVID-19. When the delta variant was reported in India, mass vaccination started worldwide. The discovery of new variants raised questions such as: How effective will vaccines be against it? [106,107]. Does vaccination reduce hospitalization and save a life? [108]. Does vaccination change transmission dynamics? [109]. Upon finding new variants, researchers started to work on the epidemiological characteristics of the variants, their transmission dynamics, reproduction number, and serial interval to find an effective strategy against them [110,111,112].

Cluster 6 (Sky Blue Cluster)

The co-occurring keywords include pandemic and outbreak. This cluster does not represent any research area or tools. COVID-19 was announced as a pandemic by the World Health Organization on 11 March 2020 [1]. Studies regarding COVID-19 often used the keyword “COVID-19”, “pandemic”, and “outbreak” together. It is visible that the “pandemic” and “outbreak” nodes have a strong link with the “COVID-19” node. Thus, this cluster is a visual representation of the outbreak of COVID-19.

## 4. Discussion

The list of top journals and studies of the intellectual structure reveals that the survey of COVID-19 transmission dynamics is a multidisciplinary topic. Among the top four journals, Science Report and PLOS One are medicine and medical-science-related work. The other two journals, Chaos, Solitons & Fractals, and Results in Physics, are pure mathematics and physics journals. This finding aligns with our intellectual network. The academic structure found that mathematicians, statisticians, and epidemiologists contributed heavily to studying COVID-19 transmission dynamics. Earlier works on COVID-19 were also the base of the COVID-19 transmission dynamics study.

Based on bibliographic data, the study tries to determine the most productive country for COVID-19 transmission dynamics. The top countries are China, the USA, the UK, India, and Canada. One interesting point could be observed from this list. Most productive countries somehow suffered severely due to COVID-19.

China was the epicenter of this disease [113]. China had to fight the virus without knowing anything about the virus. They imposed a strict lockdown and mandatory isolation policy [114,115]. It also faces political backlash from different countries.

The USA faced a different problem with its costly healthcare system, and health inequality was increasing [116,117]. Its first concern was how people would get the proper medical care required due to its expensive healthcare system. Another problem was various misinformation deliberately spread by politicians and religious and extremist groups. Many conspiracy theories were established, and different groups of people denied maintaining the COVID-19 safety protocol, further worsening the COVID-19 scenario in the USA [118,119,120].

India is the second most populated country in the world [121], with a low GDP per capita and a flawed healthcare system [122,123]. In addition, the influence of superstition is strong among Indian people [124,125]. It was a concern for researchers how a country such as India could fight this pandemic.

The co-occurrence network study reveals the research hotspots, trends, and different dimensions of the COVID-19 transmission dynamics. The network analysis provides a detailed list of mathematical tools for modeling COVID-19 transmission dynamics—these tools are used by mathematicians, epidemiologists, or infectious diseases experts. The agent-based models, SIER interval, and numerical simulations are used to model transmission dynamics. Techniques such as stability analysis are used to check the robustness of these models. Optimal control theory and basic reproduction numbers are used to make proper strategies and timely implications.

Children received special attention during COVID-19 research. Clinical characteristics of COVID-19 in children, their role as an asymptomatic spreader, and transmission dynamics in the presence of children are vital issues in this area. Studies have found that in the presence of children, transmission dynamics of COVID-19 do not change. They are not dangerous spreaders of the virus. The decision to reopen the school created another branch of research. The transmission dynamics of COVID-19 in household and family clusters have been studied to determine the effect of transmission dynamics if schools reopen [71]. Some studies have suggested that vaccinating children and adolescents can reduce COVID-19 morbidity and mortality among the population [67].

The network shows us the areas that received more attention and are growing research areas, such as “Non-pharmaceutical Interventions”. It turned out to be a specific area of study that focuses on preventing the transmission of COVID-19. Research in this area found that a combination of isolation and quarantine could be effective in preventing the spread of COVID-19. Social distancing has been proven to slow down the transmission speed. Studies regarding the effectiveness of contact tracing as a prevention strategy shows mixed results. Some studies have suggested that it is an effective strategy; some have suggested that it is ineffective.

Studying different variants of COVID-19, such as analyzing their clinical and epidemiological characteristics, their ability to break down vaccines, and transmissibility, became another study area under COVID-19 transmission dynamics analysis. There are a large number of works on the Delta variant of COVID-19. It was first reported in India. Its reproduction number, transmission dynamics, ability to break down vaccines, and vaccine effectiveness against this variant have become the research focus.

## 5. Conclusions

Our study found five distinct sub-research areas that evolved under COVID-19 transmission dynamics: (i) Tools to study COVID-19 transmission dynamics. (ii) Study of COVID-19 in Children. (iii) Epidemiology. (iv) Non-pharmaceutical interventions to prevent COVID-19. (v) Study of delta variant. These five areas are studied rigorously under COVID-19 transmission dynamics research. This study will allow future researchers to obtain quick and hands-on information about the latest research and trends and explore the possible research gaps to formulate new research projects. This study also suggested potential sources and papers that deals with COVID-19 transmission dynamics. Furthermore, the intellectual structure presented by this study also helps future researchers to understand how COVID-19 transmission dynamics research was developed.

One fundamental limitation of this study is that it was conducted using only one citation database. There are a few more databases, such as the Web of Science and PubMed. This study may be extended by including these databases.

Many papers use bibliometric analysis to conduct a literature review on COVID-19. However, no review article specifically focusing on its transmission dynamics exists. This paper fills this gap in the literature review of COVID-19, the deadliest epidemic of our time. A possible extension could be made by conducting a thorough review of the literature on mortality, vaccination, and other socioeconomic studies related to children and their well-being during the pandemic.

## Figures and Tables

**Table 1:** Types of Documents in the Sample.

Types of Document	Number
Article	933
Book Chapter	17
Conference Paper	50
Conference Review	4
Editorial	2
Erratum	5
Letter	10
Note	11
Review	70
Short survey	1
Total	1103

Source: Authors’ computation.

**Table 2:** Subject Areas of Disciplines.

Subject Area	Number of Publications *	Percentage of Publications
Medicine	532	28%
Mathematics	226	12%
Biochemistry, Genetics, and Molecular Biology	143	8%
Engineering	130	7%
Immunology and Microbiology	130	7%
Computer Science	116	6%
Multidisciplinary	101	5%
Physics and Astronomy	99	5%
Environmental Science	75	4%
Agricultural and Biological Sciences	58	3%
Others	276	15%

Note: * Many of the publications have been assigned in multiple subject areas. Source: Authors’ Compilation from the Scopus Database.

**Table 3:** Most Relevant Sources (sorted by number of articles).

Rank	Sources	Number of Articles	H-Index *	G-Index *
1	Scientific Reports	30	8	14
2	PLOS One	27	8	15
3	Chaos Solitons And Fractals	23	15	20
4	Results In Physics	23	6	12
5	International Journal Of Environmental Research And Public Health	22	8	14
6	Infectious Disease Modelling	19	8	15
7	International Journal Of Infectious Diseases	19	8	13
8	Nature Communications	16	9	14
9	Frontiers in Public Health	14	3	8
10	BMC Infectious Diseases	12	5	8

* H-index and G-index show the local impact of the journals. Source: Authors’ computation.

**Table 4:** Most Cited Papers.

Rank	Authors	Title	Total Citations	Source
1.	Chinazzi M	The effect of travel restrictions on the spread of the 2019 novel coronavirus (COVID-19) outbreak	1558	Science
2.	Kissler Sm	Projecting the transmission dynamics of SARS-CoV-2 through the post-pandemic period	1289	Science
3.	Hellewell J	Feasibility of controlling COVID-19 outbreaks by isolation of cases and contacts	1274	Lancet Global Health
4.	Kucharski Aj	Early dynamics of transmission and control of COVID-19: a mathematical modelling study	1209	Lancet Infectious Diseases
5.	Prem K	The effect of control strategies to reduce social mixing on outcomes of the COVID-19 epidemic in Wuhan, China: a modelling study	1055	Lancet Public Health
6.	Davies Ng	Estimated transmissibility and impact of SARS-CoV-2 lineage B.1.1.7 in England	854	Science
7.	Wu Jt	Estimating the clinical severity of COVID-19 from the transmission dynamics in Wuhan, China	646	Nature Medicine
8.	Cowling Bj	Impact assessment of non-pharmaceutical interventions against coronavirus disease 2019 and influenza in Hong Kong: an observational study	524	Lancet Public Health
9.	Gatto M	Spread and dynamics of the COVID-19 epidemic in Italy: Effects of emergency containment measures	504	PROC NATL ACAD SCI U S A
10.	Lavezzo E	Suppression of a SARS-CoV-2 outbreak in the Italian municipality of Vo’	502	Nature

Source: Authors’ computation.

**Table 5:** Most Productive Authors.

Rank	Authors	Number of Articles	Articles Fractionalized	H-Index *
1	Wang X	26	3.25	8
2	Li Y	18	2.29	10
3	Wang J	18	4.19	5
4	Wang Y	18	3.22	7
5	Xiao Y	17	3.17	7
6	Shah K	16	3.55	11
7	Wu J	16	1.97	6
8	Cowling Bj	15	1.82	9
9	Jit M	15	0.95	8
10	Liu Y	14	1.74	7

* H-index shows the local impact of authors. Source: Authors’ computation.

**Table 6:** Keyword Co-occurrence Network.

Cluster 1 (Red)	Cluster 2 (Green)	Cluster 3 (Deep Blue)	Cluster 4 (Yellow)	Cluster 5 (Purple)	Cluster 6 (Sky Blue)
*Tools for Studying Transmission dynamics*	*Study of Children in COVID-19*	*Epidemiology*	*Non-pharmaceutical interventions to prevent COVID-19*	*Study of delta variant*	*Outbreak of COVID-19 pandemic*
agent-based model	children	compartmental models	contact tracing	delta variant	outbreak
basic reproduction number	China	COVID-19 pandemic	isolation	India	pandemic
COVID-19 transmission dynamics	COVID-19	effective reproduction number	modelling	reproduction number	
epidemic model	COVID-19 disease	epidemiology	non-pharmaceutical intervention	serial interval	
mathematical models	epidemics	infectious diseases	quarantine	vaccination	
numerical simulations	seir model	mathematical modelling	social distancing		
optimal control	transmission	public health			
parameter estimation	transmission models	transmission dynamics			
sensitivity analysis	vaccination				
stability					
stability analysis					
quarantine					
social distancing					

Source: Authors’ computation.

## Data Availability

The data presented in this study are openly available in FigShare at https://doi.org/10.6084/m9.figshare.21429351, reference number [126].
